# Query-seeded iterative sequence similarity searching improves selectivity 5–20-fold

**DOI:** 10.1093/nar/gkw1207

**Published:** 2016-12-06

**Authors:** William R. Pearson, Weizhong Li, Rodrigo Lopez

**Affiliations:** 1 Dept. of Biochemistry and Molecular Genetics, University of Virginia, School of Medicine, Charlottesville, VA 22908, USA; 2 European Bioinformatics Institute, EMBL Outstation, Wellcome Trust Genome Campus, Hinxton, Cambridge CB10 1SD, UK

## Abstract

Iterative similarity search programs, like psiblast, jackhmmer, and psisearch, are much more sensitive than pairwise similarity search methods like blast and ssearch because they build a position specific scoring model (a PSSM or HMM) that captures the pattern of sequence conservation characteristic to a protein family. But models are subject to contamination; once an unrelated sequence has been added to the model, homologs of the unrelated sequence will also produce high scores, and the model can diverge from the original protein family. Examination of alignment errors during psiblast PSSM contamination suggested a simple strategy for dramatically reducing PSSM contamination. psiblast PSSMs are built from the query-based multiple sequence alignment (MSA) implied by the pairwise alignments between the query model (PSSM, HMM) and the subject sequences in the library. When the original query sequence residues are inserted into gapped positions in the aligned subject sequence, the resulting PSSM rarely produces alignment over-extensions or alignments to unrelated sequences. This simple step, which tends to anchor the PSSM to the original query sequence and slightly increase target percent identity, can reduce the frequency of false-positive alignments more than 20-fold compared with psiblast and jackhmmer, with little loss in search sensitivity.

## INTRODUCTION

Protein similarity searching is central to interpreting genome sequence data. The widely used BLAST program (1) can routinely identify homologous proteins that diverged >2 billion years ago, and share as little as 20–25% sequence identity. For most large, well-characterized protein families, a single BLAST search against a comprehensive protein library will yield hundreds, if not thousands of statistically significant similarity scores from homologous proteins that share common 3D structures, and often similar functions. But, despite the enormous growth in protein sequence databases, and the expectation that most protein families that exist in nature have homologs in current protein sequence databases, there are still large numbers of proteins for which little or no structural and functional information is known. Likewise, as the number of proteins with known 3D structures has grown, there are still many examples of structurally similar proteins that do not share statistically significant similarity in a BLAST search.

Iterative, model-based, similarity search methods like psiblast (1) and jackhmmer (2) are dramatically more sensitive than conventional pairwise similarity searching methods at identifying homologous, structurally similar, proteins. Iterative similarity searches with psiblast are usually two- or three-fold more sensitive than single sequence searches (3,4), and iterative methods can be 5–100-fold more sensitive with challenging queries.

Unfortunately, iterative search methods can fail when unrelated sequences are included in the Position-Specific Scoring Matrix (PSSM) or Hidden Markov Model (HMM). In the worst cases, contaminating non-homologous sequences can shift the PSSM away from the original homolog family causing it to detect more non-homologs (false-positives) than homologs (true positives). In previous work, Gonzalez and Pearson (5) showed that PSSMs are often contaminated when a homologous alignment over-extends into a non-homologous region and brings additional non-homologous domains into the PSSM model. In that work, we also showed that reducing ‘alignment creep’ by fixing the alignment boundaries for a sequence included in the PSSM to the boundaries found at the first significant alignment of the sequence, could dramatically reduce alignment over-extension and improve search selectivity (5). An implementation of this strategy—psisearch—was described by Li *et al.* (6).

During the development of psisearch2, an improved version of psisearch, we found that the strategy used to construct the PSSM in psisearch occasionally produced PSSMs that aligned incorrectly to the homologous domain. To correct this problem, we explored methods to correctly ‘anchor’ the PSSM by replacing gapped positions in the subject sequence alignment with the residues from the query sequence that aligned to the gap in the subject sequence. We were surprised to find that PSSMs constructed using query-seeded subject sequences not only reduced PSSM misalignment, they also produced dramatically fewer false-positives.

We compared the ability of conventional psiblast (1,7) and jackhmmer (2), and query-seeded versions psisearch2 using either psiblast or ssearch as the search program to identify homologs in RPD3, a set of full length protein sequences selected from Pfam28. To simulate searches with multi-domain proteins, queries were constructed by embedding Pfam domain regions from real protein sequences into random flanking sequence. We also searched with intact full-length proteins containing the same query domains. In psisearch2 searches with either psiblast or ssearch, PSSMs derived from subject sequences with seeded query residues are much less likely to become contaminated by non-homologous domains. Query-seeding appears to reduce homologous over-extension by reducing the evolutionary ‘depth,’ or increasing the target sequence identity, of PSSM models.

## MATERIALS AND METHODS

### Evaluation datasets

psiblast, jackhmmer and psisearch2 iterative search performance was evaluated using domains and sequences selected from an updated version of the RefProtDom dataset (8), RPD3, derived from Pfam release 28 (9), with modifications described below. The query sequences used for the searches contained a single Pfam28 domain, embedded in an equal length of random sequence. Query sequences were iteratively searched against the full-length protein sequences in RPD3.

#### RPD3 construction—selection of domain families and clans

The domain families used to evaluate iterative similarity search strategies were selected from RefProtDom3, a set of diverse domains families that met the following criteria: (i) domain size: Pfam domain model lengths >200 match states; (ii) domain number: >200 members; (iii) diversity: domains were present in at least two of the three kingdoms of life (archaea, bacteria, eukaryota) with at least 20% of the sequences from the second most abundant kingdom (100 domains if the most abundant kingdom had >500 domains); (iv) clan length consistency: domain families from clans were included only if the maximum model length of the domains in the clan was <1.5-fold the minimum model length. In Pfam28, there are 1743 domain families and 155 clans that met the domain length, domain abundance, and clan length consistency criteria. Including the diversity requirement reduced the number of domain families that did not belong to a clan to 299, and the number of clans to 40, for a total of 339 non-homologous queries from 428 Pfam28 domain families.

#### RPD3 construction—selection of sequences

With the dramatic increase in bacterial sequencing over the past 5 years, some of the 339 domain and clan RPD3 families contained many tens of thousands of sequences containing an RPD3 domain. To reduce the differences in abundance between the largest and smallest domain families, large domain families were randomly down-sampled to a maximum of 5000 entries using a strategy that sought to preserve or enhance phylogenetic diversity. Thus, if there were 2000 or fewer sequences in archaea, bacteria, and eukaroyta, all 2000 were included, and if the two kingdoms with fewer domains had <25% of the domains in the largest kingdom, 2000 were taken from the most abundant, and at least 500 taken from the less abundant kingdoms. If the less abundant kingdoms contained >25% of the sequences in the most abundant kingdom, all three kingdoms were sampled randomly. The same strategy was used for domains from the 40 clans, but for domains in clans, all the domain families from the clan were combined, and then the phylogenetic diversity rules were used for sampling.

The resulting RPD3 protein set contains 597 753 proteins that contain at least one domain from the 299 Pfam28 families and 40 Pfam28 clans in the RPD3 domain set. The largest clan/domain family is found in 4719 sequences, the smallest domain family in 207 (median: 2271, Q1: 1011, Q3: 2482). Thus, the middle 50% of clan/domain families differed ∼2.5-fold in abundance. The full length RPD3 sequences contain many domains in addition to the 339 domains/clans in the RPD3 set. Pfam28 reports 2904 domains in the full alignments (Pfam28 MySQL field in_full=1) among the RPD3 sequence set.

#### Query sequence selection

To provide a challenging set of domains for evaluating psiblast, psisearch2, and jackhmmer, two sets of 100 query domains were selected from the 339 RPD3 clan/domain families. We sought sequences that were more distant from the HMM model that describes the domain family, using the Pfam28 sequence_bits_scores as a proxy for evolutionary distance. For one set of 100 queries (far50), we randomly selected domains from the bottom tenth-percentile of sequence_bits_scores from the pfamA_reg_full_significant table in the Pfam28 MySQL distribution that covered at least 50% of the domain model length, and did not overlap other domains. For the second set of 100 queries (far66), we randomly selected domains that at least 66% of the model length from the bottom tenth-percentile. For domain families that belonged to clans, we selected a domain family near the median in sequence abundance in RPD3. Domains were only selected from sequences that were not marked as is_fragment in Pfam28.

The domain region sequences were then embedded into random sequence, i.e. a 200 residue domain produced a 400 residue query sequence with 100 residues of random sequence on either side of the genuine domain sequence, and used to search the RPD3 sequence set. The embedded domain queries were then ranked by their ability to produce statistically significant alignments in a Smith–Waterman search (ssearch) of the RPD3 library, and the 100 sequences between the 10th and 40th percentiles by family coverage were selected (far50). For the far66 set of queries, the 100 embedded domain queries were selected randomly from the bottom half of queries ranked by family coverage after an ssearch search. The two query sets sample 150 of the domain/clan families. Forty-nine families are shared (with different embedded domain queries) between the far50 and far66 query sets. We also evaluated the performance of query-seeded iterative searching using full-length protein sequence queries by retrieving the full-length sequences from Pfam28 that contained the 100 far50 embedded domains (far50-full) and the 100 full-length sequences with far66 domains (far66-full).

#### Challenging Pfam queries

To focus on the domain queries that produced the largest false discovery rates (FP/(TP+FP), FDR), we identified 40 queries with the highest FDR for psiblast and 40 for jackhmmer, and then found 20 each from the far50 and far66 sets with the highest average FDR using psiblast and jackhmmer after 10 iterations.

### Iterative searching and PSSM construction

**Figure 1.**
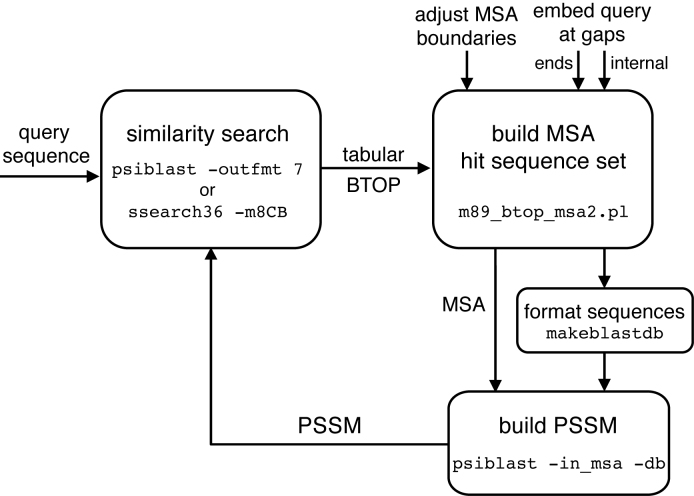
The psisearch2 iteration cycle. A query sequence is compared with a sequence database using either psiblast or ssearch, producing output in BLAST tabular format including a BTOP encoded alignment. The tabular BTOP output, together with optional boundary information, is processed by the m89_btop_msa2.pl script to produce both a multiple sequence alignment (MSA) and a FASTA format library file which is reformatted with makeblastdb. These two files are processed by psiblast to produce a PSSM, which can then be used to re-search the sequence database for the next iteration.

We evaluated the performance of psisearch2, a new version of the psisearch program (6) that combines query-seeding and alignment boundary modification. The psisearch2 script (Figure 1) separates the two parts of an iterative search: (i) the identification of homologs and production of alignments; and (ii) the production of a PSSM from the alignments for the next iteration. psisearch2 uses a flexible strategy for modifying the boundaries of the multiple sequence alignment (MSA) and the sequence library used to construct the PSSM with psiblast.

A block diagram of the search/alignment/PSSM construction process is shown in Figure 1. The iterative process begins with a similarity search, using either psiblast (7) or ssearch (10), which produces output in the blast-tabular format that includes the blast BTOP alignment encoding (psiblast-outfmt 7 or ssearch -m 8CB) format. The alignment results are passed to the m89_btop_msa2.pl script to produce a MSA and subject sequence library. The MSA and subject library files are then passed to psiblast to produce the PSSM.

We control the PSSM construction process by using psiblast2.3.0 with the -in_msa and -out_pssm options, together with the recently implemented -save_pssm_after_last_round option. We build the PSSM by aligning the MSA to a sequence database comprised of the sequences with statistically significant similarity scores from the previous iteration. We can modify the properties of the PSSM in two ways: (i) by controlling the boundaries of the sequences specified in the MSA used to make the PSSM (boundaries can reflect the current alignment boundaries, the previous alignment boundaries, or domain boundaries); and (ii) by modifying the subject sequences in the sequence database used to calculate the PSSM (both internal- and end-gaps in the subject sequence can be ignored, or be substituted with the aligned query sequence residue, or with a random residue).

The m89_btop_msa2.pl program takes the alignments produced by the psiblast or ssearch similarity search and produces an MSA and a custom subject sequence database that psiblast can convert into a PSSM. m89_btop_msa2.pl options control the MSA boundaries and the sequences in the custom subject sequence database.

### Search evaluation

#### Characterization of true-positives and false-positives

The 200 embedded domain query sequences described above were used to iteratively search the RPD3 full-length protein sequence database using psiblast and jackhmmer (unmodified), and psisearch2 modified using the query-seeding and boundary control strategies available with m89_btop_msa2.pl (Figure 1).

For the psiblast and ssearch searches with psisearch2, alignment output was captured using the commented tabular format, including the BTOP field. For jackhmmer, similar information was extracted from the –domtblout file. The blast-tabular and –domtblout formats provide both the identifier and expectation value for the subject library sequences found, and the beginning and ends of the alignments in the query and subject sequence. For jackhmmer, we used hmm coordinates as a proxy for the query coordinates, and the alignment start and end, not the probabilistic envelope boundaries, for the subject boundaries.

**Figure 2.**
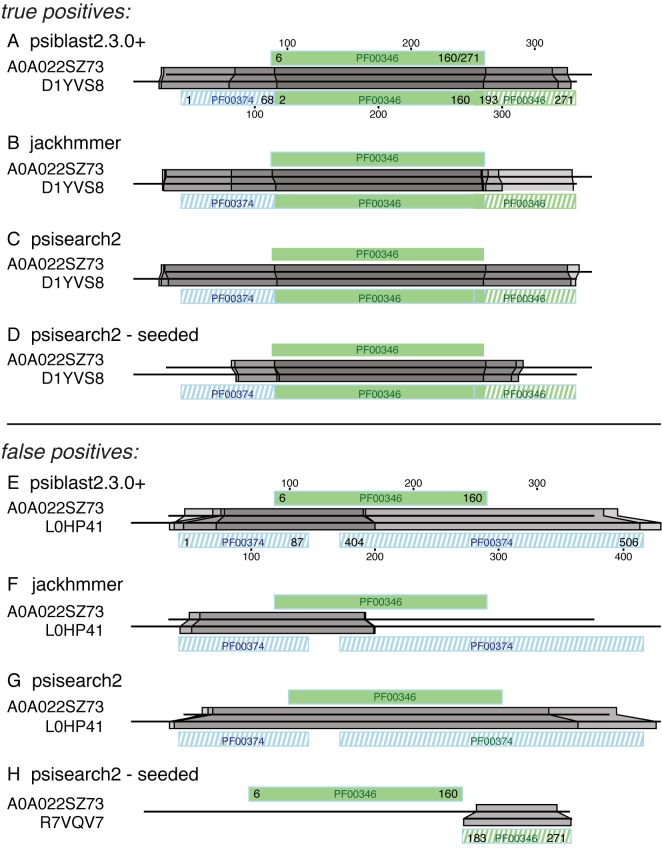
Iterative over-extension with successive iterations psiblast, jackhmmer, and psisearch. The respiratory-chain NADH dehydrogenase domain (PF00346) comprising residues 87–259 from Uniprot protein accession A0A022SZ73 was embedded in 86 residues of random sequence on the N- and C-terminal sides, and compared to the RPD3 library using the indicated programs. The two horizontal lines indicate the the query and subject sequences; the green and blue blocks above and below the alignment blocks show all Pfam28 domains on the query and subject sequences. Green domain alignments in solid colors reflect homology; striped blue and green domain alignments are non-homologous false positives. The start, end, and length of the Pfam28 model are shown in the domain blocks. The gray-shaded blocks show the boundaries of the alignments. Alignment boundaries in the initial iterations are darkest; boundaries in the final iterations are lighter. (A–D) Progress of the first five iterations in alignments to a protein (Uniprot accession D1YVS8) that contains an homologous PF00346 domain (green) with psiblast (**A**), jackhmmer (**B**), psisearch2 (**C**) and psisearch2 with query-seeding (**D**). (E–H) False-positive alignments to non-homologous regions. The first significant (**E**) < 0.001) false-positive alignment to Uniprot accession L0HP41 with psiblast (E, iteration 2), JACKMMER (**F**, iter. 3), and psisearch2 (**G**, iter. 3). psisearch2 with seeding (**H**) does not produce a significant alignment with L0HP41, so an alignment with Uniprot accession R2VQV7 at iteration 4 is shown (H). This alignment is to a homologous domain, but in a non-homologous region, so the alignment is scored as a false-positive.

Because our query sequences contain a genuine protein domain sequence embedded in random sequence, we count alignments as true-positives only if the genuine domain in the query aligns with the same domain in the Pfam28 annotated RPD3 protein sequence. If the embedded domain query sequence domain aligned with a protein that did not contain the correct domain, the alignment was scored as a false-positive. If the query and subject sequences contained the same domain, but the alignment was outside the embedded domain coordinates, the alignment was scored as a false-positive (Figure 2H). For searches with full-length proteins (far50-full, far66-full), only alignments in the original query domain were scored. Thus, for full-length query sequence H6NQX3, which contains a PF02219/CL0086 domain from residues 326–607, only alignments within this range of query residues were scored as either true-positive (if the aligned region contained a CL0086 domain), or false-positive (if no true-positive domain was found). This contrasts with embedded queries, which could be scored as false-positives when the alignment occurred in the random flanking sequence (Figure 2H).

Average overall sensitivity and FDR was calculated as weighted sensitivity (TP/(TP + FN)) or FDR (FP/(TP + FP)) for receiver operator characteristic (ROC) curves and Table 1 by treating each family independently, adding up the total sensitivity or FDR, and dividing by the number of queries. Thus, when 100 queries were summarized, each individual query contributed a maximum of 1% of the sensitivity or FDR total; for 20 queries, 5%.

**Table 1.** False discovery rates (FDR) and sensitivity (maximum family coverage) for far50 queries

Program	Iter.	F50%^a^	FDR 80%^b^	FDR Max.^c^	Sens.^d^
psiblast	1	–	–	0.0014	**0.1115**
	5	0.0001	0.0037	0.0321	0.8833
	10	0.0003	0.0112	0.0653	0.9161

jackhmmer	1	–	–	0.0097	0.1097
	5	0.0009	0.0052	0.0232	**0.8955**
	10	0.0010	0.0061	0.0357	**0.9299**

psisearch2/msa	1	–	–	**0.0011**	0.0984
	5	**0.0000**	0.0016	0.0168	0.8452
	10	**0.0000**	0.0009	0.0319	0.8931

psisearch2/msa+seed	1	–	–	**0.0011**	0.0986
	5	**0.0000**	**0.0004** 3.8X	**0.0009** 18X	0.8208
	10	**0.0000**	**0.0002** 4.9X	**0.0018** 17X	0.8626

psiblast/msa	1	–	–	0.0014	**0.1115**
	5	**0.0000**	0.0035	0.0355	0.8873
	10	**0.0000**	0.0193	0.0798	0.9139

psiblast/msa+seed	1	–	–	0.0014	**0.1115**
	5	**0.0000**	0.0006 5.9X	0.0050 7.1X	0.8518
	10	**0.0000**	0.0005 38X	0.0149 5.4X	0.8842

^a^False discovery rate (FDR) at 50% weighted family coverage after 5 or 10 iterations (Iter.).

^b^FDR at 80% family coverage.

^c^Maximum FDR.

^d^Maximum weighted family coverage. ‘X’ values show the reduction in FDR compared with unseeded MSAs for psiblast and psisearch2.

#### Modifications to Pfam28 annotations

Our evaluation of search effectiveness and search selectivity depends on accurate Pfam28 annotations. We used Pfam28 coordinate annotations on the proteins in RPD3 without modification. But when we saw significant false-positive alignments, sometimes with unembedded sequences, and sometimes in the first iteration, we investigated further. In four cases, we concluded that Pfam had missed a homology relationship. We added PF09511 to clan CL0078, PF16332 to CL0579, PF01010 to CL0425, and we formed a new clan (CL9001) from PF01156 and PF07362. Each of these relationships was confirmed by finding alignments where annotated members of the clan aligned with the candidate homologous domain, or where the relationship was supported by SCOOP (11). Adding Pfam domain families to clans allowed us to correct large numbers of false-positives (but also increased the number of homologs in the family, thus reducing the true-positive fraction). It is unlikely that we have corrected all the missing relationships, so some of the false-positives we record are probably the artifact of homologous domains that are not annotated by Pfam28.

### Software availability

The psisearch2.pl, psisearch2.py, and m89_btop_msa2.pl scripts are available as part of the FASTA software distribution from faculty.virginia.edu/wrpearson/fasta/fasta36 directory, or from the European Bioinformatics Institute (ftp://ftp.ebi.ac.uk/pub/software/psi-search2), or from GitHub (github.com/wrpearson/fasta36). The RPD3 database is available from GitHub (github.com/wrpearson/RPD3).

## RESULTS

### Alignment over-extension and PSSM corruption

PSSM corruption often occurs when an aligned homologous region produces a strong similarity score that allows the alignment to be continued into an adjacent non-homologous region, a process we have termed homologous over-extension (5). Homologous over-extension typically occurs because the alignment score for an homologous region is not reduced rapidly enough in the non-homologous region to terminate the alignment. For example, if the homologous region is around 40% identical and the BLOSUM62 scoring matrix is being used, the alignment might be extended into non-homologous until the overall alignment identity is 25% or lower (12). Homologous over-extension is a particular problem for iterative methods that build protein family specific PSSMs or HMMs, because these scoring systems can detect very low identity homologs, and are thus less effective at terminating alignments as they extend into non-homologous regions.

When we first identified homologous over-extension as a major cause of PSSM contamination, we found that we could reduce contamination using a simple strategy to prevent alignments between the query/PSSM and the subject sequences from extending with successive alignments (5,6). This strategy improved search specificity but seemed crude, since distantly related portions of an homologous region that failed to align in an early iteration might be excluded from the PSSM. Thus, we sought a more subtle strategy for reducing over-extension.

Recently, the fasta programs have been extended to allow sub-alignment scoring (10), a process that partitions the overall similarity score based on sequence annotations, such as the start and stop of domains annotated by Pfam (9). Sub-alignment scoring makes it much easier to detect potential non-homologous alignment, because the part of the alignment that is homologous will have a much higher score than the over-extended non-homologous region. When non-homologous over-extension occurs, more than 80% of the similarity score can be found in the homologous region, but the non-homologous alignment with 20% of the score may be from 10–100 residues long. Thus, the score density in the non-homologous region is far lower than the density across the homologous alignment. Sub-alignment scoring can detect domains that have been included in alignment but do not contribute significantly to its score.

Our earlier psisearch program (6) performed iterative searches by searching a database (upper left box in Figure 1) and then directly building a PSSM by running psiblast with the query sequence or query PSSM against a library of subject sequences produced from the significant alignments in the previous search (lower right box in Figure 1). While integrating sub-alignment scoring into the scripts that we used for iterative searching with ssearch, we were surprised to find that on rare occasions the psiblast run to produce the PSSM did not align the query/PSSM to the same region of the subject sequence. To provide psiblast more guidance and ensure that the appropriate sequences were aligned, we wrote the m89_btop_msa2.pl script. m89_btop_msa2.pl produces an MSA from the aligned output of the previous search. psiblast can use this MSA, together with the set of subject sequences (the two sets of arrows entering the “Build PSSM” box in Figure 1) to produce a PSSM. But despite the MSA input, psiblast sometimes failed to produce an alignment over the homologous domain.

To ‘force’ psiblast to accurately reproduce the alignments over the homologous domains, we modified the m89_btop_msa2.pl script to insert query residues into the subject library sequences at positions corresponding to gaps in the subject sequence in the MSA alignment. This strategy abolished psiblast misalignment during PSSM generation, and we were to find that it also dramatically reduced the number of false-positives found after five, or even ten, iterations. Including ‘X’-residues, or random residues, did not consistently prevent misalignment.

Figure 2 shows the process of alignment over-extension that occurs when a sequence with a Pfam28 domain (PF00346) embedded in random sequence aligns with full-length Uniprot proteins. The PF00346 domain from A0A022SZ73 embedded in the query comes from the far50 query set; each of these domains must cover at least 50% of the Pfam28 model. The A0A022SZ73 domain contains 155 (6–160) of the 271 match state HMM PF00346 model. This incomplete domain can produce alignments with other PF00346 containing proteins that extend into the random C-terminal sequence.

All the alignment beyond the embedded green PF00346 domain in Figure 2 is non-homologous over-extension; outside the green domain the subject sequence is aligning to random sequence in the query (indicated by striped domains in the subject sequences). As Figure 2, panels A–D illustrate, over-extension occurs with NCBI psiblast, jackhmmer, and psisearch2 (without query-seeding), and with psisearch2 with query seeding to a more limited extent. Many proteins that contain a PF00346 domain also contain a PF00374 domain (e.g. L0HP41 in Figure 2A–D). When over-extension aligns random sequence in the query to a PF00374 domain in the subject sequences, the PSSM ‘learns’ to find PF00374 domains, and produces false-positive alignments with proteins that contain a PF00374 domain, but not the homologous PF00346 domain (Figure 2E–G, striped blue domains). If the PSSM is built by aligning the MSA against a library that contains query residues seeded in the gaps in the subject sequences, the PF00374 alignment does not occur, though it does align to a non-homologous part of a PF00346 domain (Figure 2H, striped green domain).

### Query-seeding reduces false-positives

Query-seeding reduces the sensitivity of psisearch2 and psiblast slightly, but decreases the false discovery rate (FDR) for those iterative methods 5–20-fold (Table 1, Supplementary Data, and Supplementary Data). The 100 far50 queries are quite challenging—all the methods find about 10% of homologs after the first iteration (for the far66 embedded queries, ∼15% of homologs are found after one iteration)—but after five or ten iterations, 82–93% of homologs (the average across the 100 queries) are found. jackhmmer detects the largest fraction of homologs, but also has the highest average FDR.

The more remarkable difference in performance with query-seeding is reflected in FDR after five and ten iterations. The direct effects of query-seeding can be seen by comparing the psisearch2/msa and psisearch2/msa+seed, or the psiblast/msa and psiblast/msa+seed maximum FDR columns. For these two pairs, the only difference in how the PSSM was constructed and used was the inclusion of query residues in subject sequence gaps.

With the far50 embedded domains, query-seeding drops the maximum FDR for psisearch2 18-fold after five or ten iterations (Table 1). For psiblast, query-seeding improves the FDR 7-fold after five iterations and 5-fold after 10 iterations. For the far66 queries (Supplementary Data), query-seeding reduced the maximum FDR about 5-fold. At 80% average family coverage, query-seeding improved FDR from 4–38-fold for the far50 queries. For the far66 queries, the 80% FDR improvement ranged from 38-fold (psisearch2) to 79-fold (psiblast) at iteration 10 (Supplementary Data).

After 10 iterations, 36 far50 queries produced a false-positive with unseeded psisearch2, but only 16 produced a false-positive with query-seeded psisearch2. Similar results occur with MSA driven psiblast (Figure 1). After 10 iterations, 54 queries produce a false positive with psiblast/MSA, but only 32 queries produce a false-positive with psiblast/MSA with query-seeding.

**Figure 3.**
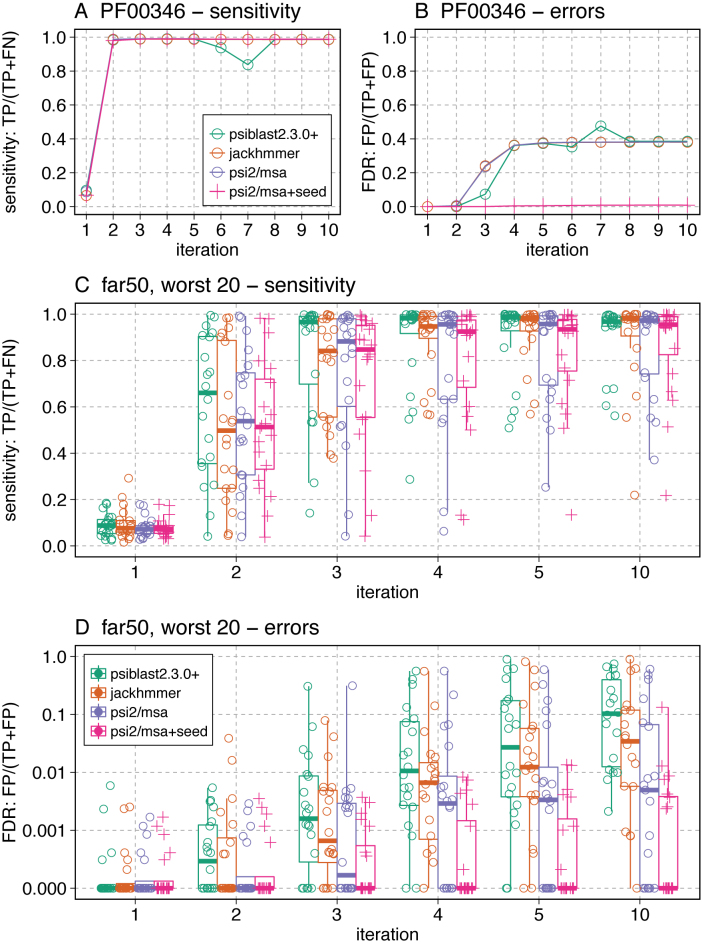
Sensitivity and selectivity (FDR) for PF00346 (**A, B**) and 20 challenging far50 queries (**C, D**). The distribution of the sensitivity (A, C; fraction of true positives found) and FDR (B, D) are shown for four different search strategies, NCBI psiblast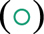, jackhmmer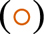, psisearch2 without query-seeding 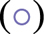, and psisearch2 with query-seeding 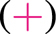. The boxplots show the median, first and third quartiles, and 1.5 times the inter-quartile range. FDR (panel D) is plotted on a log scale. The median bar is not visible in the psisearch2 seeded box because fewer than half of the challenging families have false-positives. Data for the far66 query set ares shown in Supplementary Data.

The far50 and far66 queries were selected because they share significant similarity with the smallest fraction of homologs in the RPD3 database. But half of the far50 queries produce no false-positives after five iterations with psiblast (Supplementary Data), and more than one third of the far50 queries produced no false-positives after 10 iterations with psisearch2 (un-seeded). Thus, we focus on the 20 families from the far50 and far66 query sets that produced the largest FDR (Figure 3, Supplementary Data).

Figure 3 shows the sensitivity and selectivity (FDR) of PF00346 (panels A and B) and twenty of the most challenging query sequences from the far50 query set (the far66 dataset is shown in Supplementary Data). At iteration 2, psiblast produces 15 false-positive alignments in addition to finding 98% of the 2761 true-positives, while psisearch2 (unseeded) produces six false-positives. jackhmmer reports its first 217 false-positives with PF00346 at iteration 3 (Figure 3). At iteration 4, jackhmmer produces 1541 false-positives. psisearch2 with seeded query residues produces 1 false-positive at iteration 3, but only 24 after 10 iterations, where the non-seeded iterative strategies produce ∼1700 false-positives.

**Figure 4.**
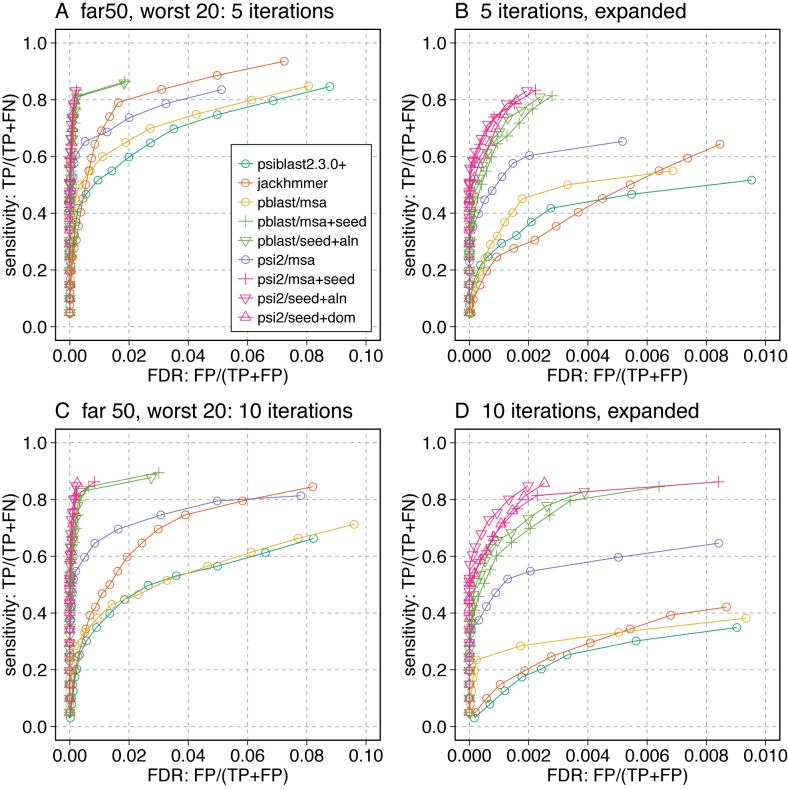
Seeding and over-extension control strategies. Nine iterative search strategies are shown on the 20 most challenging queries from the far50 set. In addition to the six strategies shown in Figure 3, three combinations of two over-extension limiting strategies are shown: (i) psiblast/msa with alignment extension limited by alignment history (pblast/seed+aln, 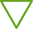); (ii) psisearch2 with seeding limited by alignment history (psi2/seed+aln, 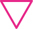) and (iii) psisearch2 seeded with domain boundaries (psi2/seed+dom, 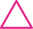). Results after five (**A, B**) or ten (**C, D**) iterations. Plots for the far66 dataset are shown in Supplementary Data.

The process of PSSM contamination depends strongly on the content of the sequence database and the topologies of the homologous and non-homologous domains. For a broader perspective on search performance we plotted the true positive fraction and FDR for the 20 hardest far50 queries (Figure 3C and D). Here, the median FDR shows that psiblast begins producing false-positives for more than half the queries by iteration 2, and jackhmmer and psisearch2 (unseeded) at iteration 3. psisearch2 (seeded) does not produce any false-positives for half the queries in the far50 query set after 10 iterations. In the far66 query set, more than half the queries are producing false-positives with psisearch2 (seeded) at iteration 4, but the FDR is an order of magnitude lower than psisearch2 without query-seeding (Supplementary Data).

We also compared the performance of PSSMs produced with and without query-seeding by simply tabulating the number of query families where the seeding produced more false-positives, or fewer false positives using the ‘R’ binom.test function. This test confirms that query-seeding significantly reduces the number of false-positives. For psiblast and the far50 data set, 46 queries produce more false-positives after 10 iterations, while 16 queries produce fewer (*P* < 10^−4^, ‘R’ binom.test, one-tailed). With the far66 dataset, the numbers are 55 more and 4 fewer false-positive queries without query-seeding (*P* < 10^−12^). For psisearch2, 38 families have more false-positives and nine fewer on the far50 dataset (*P* < 10^−5^), while 36 have more and five fewer on far66 (*P* < 10^−6^). When the same test is done on the number of true positives, query-seeding reduces sensitivity. For psisearch2 and the far50 set, unseeded PSSMs perform better with 63 queries, while query-seeded PSSMs perform better with 32 (*P* < 0.001). But we believe that false-positives pose much more of a threat to iterative searches than false-negatives (see Discussion). Query-seeding significantly reduces the number of false-positives during iterative searches with only a small decrease in sensitivity.

### Controlling alignment extension reduces over-extension

The improvement we see by seeding query residues into gaps in subject sequences is larger than the improvement we found by explicitly limiting alignment extension in psisearch (6). To see whether additional control of alignment extension could improve FDR beyond query-seeding, we examined two alignment strategies for reducing over-extension: (i) setting alignment boundaries to the values found the first time the subject sequence was found with a statistically significant score (alignment) (5,6); and (ii) limiting alignment extension to domain boundaries based on Pfam28 domains (domains). Limiting extension based on alignment history was tested using both psiblast/MSA and psisearch2 (both with seeding). Domain based extension limits only tested with psisearch2, since the approach uses sub-alignment scores to focus on domains with significant similarity.

**Figure 5.**
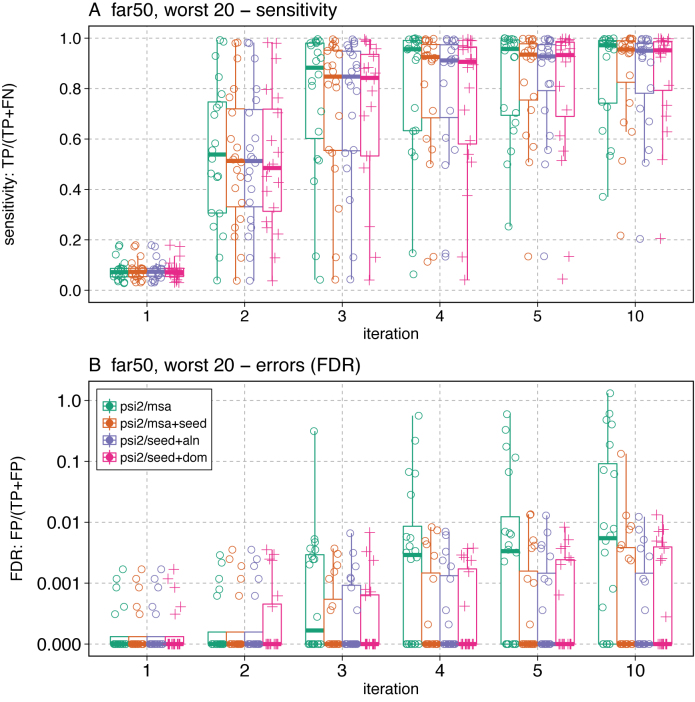
Alignment boundary modification modestly improves selectivity. The sensitivity (**A**) and FDR (**B**) for each of the 20 challenging queries from the far50 set are shown for four variations of psisearch2: without query-seeded PSSMs 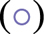, with query-seeded PSSMs 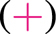, query-seeded PSSMs with over-extension limited by alignment history (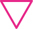) or domain boundaries (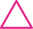). The boxplots and data points are drawn as in Figure 3.

**Figure 6.**
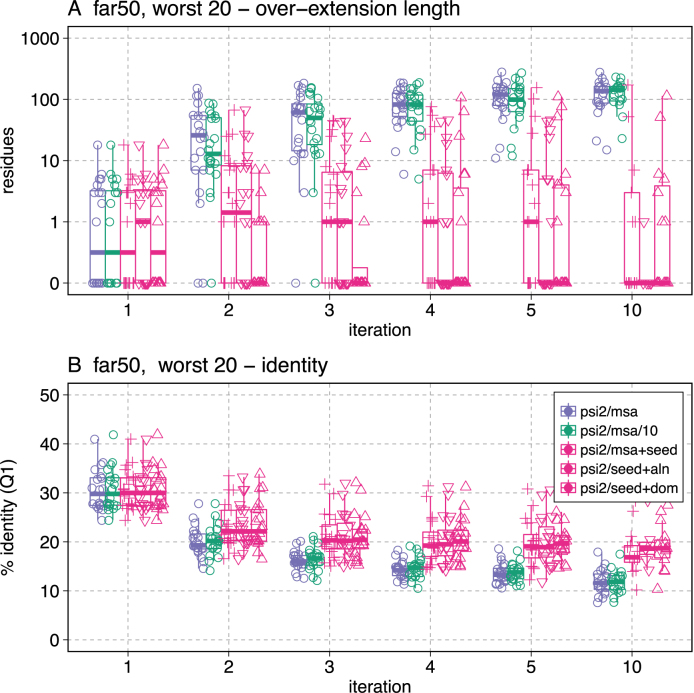
Query-seeding reduces over-extension and increases alignment identity. The distributions of the median over-extension (**A**) and the bottom quartile of percent identity (**B**) for all alignments with *E*() < 0.001 for the 20 challenging far50 queries is shown. Symbols and colors are as in Figure 5, with the addition of psi2/msa/10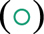, which shows the alignment progress with psisearch2, without query-seeding, but with a 20-fold more stringent inclusion threshold (–evalue=0.0001). The same data for the far66 queries are shown in Supplementary Data.

In our tests, limiting alignment over-extension using the alignment history was generally more effective than the domain strategy (Figures 4, 5 and 6A). Looking at the ROC curve (Figure 4) the up- and down-triangles (▵, ▽) both produce curves to the left (more selective) of the curves with seeding alone (+ symbols) after 10 iterations. But the effect is quite modest. Looking at the distribution of FDR fractions across 10 iterations (Figure 5 B) suggests that the alignment history strategy does a better job of controlling the FDR for more of the 20 hardest queries.

Limiting alignment extension using alignment history has a small effect on psiseach2 with query-seeding and the far50 and far66 datasets; the number of false-positives goes down for seven families but up for four for the far50 queries, and down for eight but up for three with the far66 set. The effect for psiblast is more dramatic; 19 families have fewer false-positives with using alignment history to reduce over-extension, while eight families have more false-positives (*P* < 0.026 using a one-sided binom.test function in ‘R’). For the far66 set, 17 families have fewer false-positives and six have more (*P* < 0.017).

Because query-seeding is very effective, we only see a modest improvement in search selectivity (FDR) with our two over-extension control strategies. Figure 6A shows how the amount of over-extension increases with number of iterations when query-seeding is not used 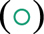. Remarkably, when query-seeding is used, the median level of over-extension without alignment boundary modification is <10 residues; query-seeding reduces median over-extension more than 50-fold after five and ten iterations. With so little over-extension without alignment boundary modification, it is difficult to do much better, and domain-based boundary modification looks very similar to query-seeding alone. However, boundary modification using alignment history reduces over-extension even more, particularly after iteration three.

Seeding query residues into the sequences used to construct PSSMs reduces false-positives by increasing the information content at positions with gaps in many homologs, effectively making the resulting position specific scoring matrix slightly ‘less deep’. But query-seeding also reduces failures of the PSSM construction process to re-align sequences properly. To see whether query-seeding improves searches where over-extension is less likely, we also compared the unseeded domains from the far50 query set to the RPD3 database. When the far50 domains are not embedded in random sequence, there were no significant differences between un-seeded and seeded PSSM searches (Supplementary Data), and, as expected PSSMs built with query seeding are slightly less sensitive.

### Query-seeding and PSSM target identity

Homologous over-extension, the major cause of false-positives in iterative searching, can be reduced by decreasing the evolutionary distance (equivalent to increasing the target percent identity) of the scoring matrix used to produce alignments (12,13). Query-seeding increases the median percent identity of the bottom quartile of alignments at iteration five and ten by about 5% identity (e.g. from 13.3% (far50) to 19.0%, Figure 6B). Overextension does not cause reduced target identity; higher identity with seeding occurs with non-embedded searches, which cannot over-extend.

To test whether the higher sensitivity of the matrices constructed from unseeded alignments contributes significantly to alignment over-extension, we reduced search sensitivity by specifying a 10-fold lower *E*()-value, 0.0001 rather than 0.002 (the default), for inclusion in the MSA and PSSM (Figure 6, psi2/msa/10). The more stringent inclusion threshold is less sensitive than query-seeding with *E*() < 0.002, but it does not substantially affect either the amount of over-extension or the target percent identity (Figure 6). Thus, we believe that the higher information content of the scoring matrix, rather than the reduced sensitivity of the search, limits over-extension. Query-seeding effectively makes the PSSMs less evolutionarily ‘deep,’ which produces higher identity alignments (14) and less homologous over-extension (12,13).

### Iterative searches with full-length proteins

Query-seeding dramatically improves search selectivity with embedded queries (Figures 3–5, Table 1, Supplementary Data) by reducing alignment over-extension (Figure 6). But because they are surrounded by random sequence, our embedded queries encourage alignment over-extension.

Query-seeding also improves search selectivity in with unembedded, full-length, protein sequences (Supplementary Data). For the far50 full-length proteins, query-seeding reduced maximum FDR 2–6-fold after five and ten iterations. For the far66 full-length proteins, query-seeding reduces FDR 1.4–3.7-fold at 80% coverage, and 3–4-fold at maximum coverage. Query-seeding reduced sensitivity about 5%, somewhat >2–3% reduction seen in Table 1 and Supplementary Data. Full-length query sequences have a lower FDR than embedded domains, but query-seeding can provide an additional improvement.

## DISCUSSION

Iterative sequence similarity searching with psiblast began with the observation by Henikoff and Henikoff (15) that position based sequence weights (PSSMs) embedded into the conserved regions of a query sequence are dramatically more sensitive than searching with the sequences alone. This strategy, implemented in the COBBLER program, provided the basis for psiblast (1), which revolutionized sequence similarity searching by exploiting conservation information in sequence databases to dramatically increase the sensitivity of sequence searches. Subsequent improvements in psiblast have improved performance by more robustly dealing with composition bias and using more sophisticated methods to initialize the PSSM (16–20). These strategies have improved psiblast performance when identifying homologs, but the same strategies that allow the identification of more distant relationships—the construction of more sensitive PSSMs—also increase the likelihood of alignment over-extension (5,12).

In this paper, we demonstrate that the rediscovery of the Henikoffs’ original observation (15), that PSSMs can be embedded in a query sequence, can dramatically reduce false-positives in iterative search strategies. The simple strategy we developed and implemented in the m89_btop_msa2.pl script can be combined with either psiblast or psisearch to construct PSSMs that are less likely to produce homologous over-extension. Using query-seeded PSSMs, the number of false-positives drops from over a thousand to fewer than a dozen with some queries.

While the weighting of residues and pseudo-counts to construct better PSSMs has been examined very carefully, there is much less information available about how to treat gaps when constructing a PSSM. Gaps tend to be clustered in the MSA and may be indicative of less well-conserved regions. Inserting query sequence residues back into the gapped positions in subject sequences to build the PSSM is complementary to the original Henikoff seeding strategy, which embedded the PSSM in the query.

Query sequence seeding reduces alignment over-extension by increasing the information content at gapped positions in the MSA that is used to construct the PSSM. This increased information content shifts the PSSM target identity to a shorter evolutionary distance (Figure 6B), which tends to reduce over-extension (12). Since the gaps in the MSA are often found near the ends of the homologous domain, the mismatch penalties near the ends of the domain boundaries are also increased, which reduces the likelihood of over-extension.

Modifying the PSSM by seeding query residues dramatically reduces false-positives, but it also slightly reduces true positives (Table 1). On the most challenging far50 query set, our most effective strategy for reducing false-positives reduces sensitivity after 10 iterations from 93.9% (jackhmmer) to 86.9% (psisearch2/seed+aln), while reducing the FDR from 13.9% to 0.2%. On the far66 dataset, sensitivity drops from 93.0% to 88.3% while the FDR drops from 25.9% to 3.5%. Thus, 5–10% drops in sensitivity yield 8–20-fold, or more, reductions in FDR.

We believe that the modest decrease in search sensitivity is more than balanced by 10-fold reductions in FDR. As Pfam clans illustrate, for many large and diverse protein families it is not possible to build a single model, either PSSM or HMM, that can reliably detect all the members of the family. In Pfam30, about one-third of Pfam domain families belong to clans, but even with multiple models in clans representing a single domain family, many Pfam domain homologs remain unannotated. Complete identification of homologous domains requires a mixture of PSSM or HMM models and a strategy for re-starting the iterative search to build a new homologous model. Such transitive strategies are far less likely to become contaminated if false-positives are avoided.

Most of our false-positives with query-seeded PSSMs align to the query domain, not to the random sequence surrounding the embedded domain. We cannot be certain that the false-positives that we find with our most selective methods are genuine non-homologs; some are likely to be cryptic homologs. If homologous over-extension can be eliminated with some combination of PSSM adjustment and alignment boundary modification, then the problem of false-positive detection becomes statistical, and it should be possible to develop better methods with even fewer errors.

## CONCLUSION

Improvements in sequence similarity searching require more sensitive scoring matrices—evolutionarily ‘deep’ matrices that can detect homologs with low sequence identity by giving low-identity alignments positive alignment scores. But matrices that give positive scores to low identity homologs are also much more likely to allow alignments from homologous domains to extend into non-homologous regions. Iterative searching is effective because the discovery of one or two distant homologs can reveal hundreds of new homologs in the next iteration. But this same amplification process makes it critical to avoid false-positives; one or two false-positive relationships can quickly lead to hundreds or thousands of misleading results. Query sequence seeding dramatically reduces the incidence of false-positives. With psisearch2 query-seeding and either alignment or domain boundary limits, more than half of our most challenging queries do not produce any false-positives, even after 10 iterations. The higher selectivity of query comes at a cost of slightly lower sensitivity, which can be partially offset by increasing the number of iterations, but alternative strategies, such as re-initiating the search with a distant homolog, may be required to identify the most distant homologs.

## Supplementary Material

Supplementary Data
