# Supplementary material for: Query-seeded iterative sequence similarity searching improves selectivity 5–20-fold
Source: Nucleic Acids Res. 2016 Dec 6;45(7):e46. doi: 10.1093/nar/gkw1207 (PMC5605230; doi:10.1093/nar/gkw1207)
Supplement: Supplementary Data [file gkw1207_supplementary_data.zip › nar-02847-met-n-2016-File011.pdf]

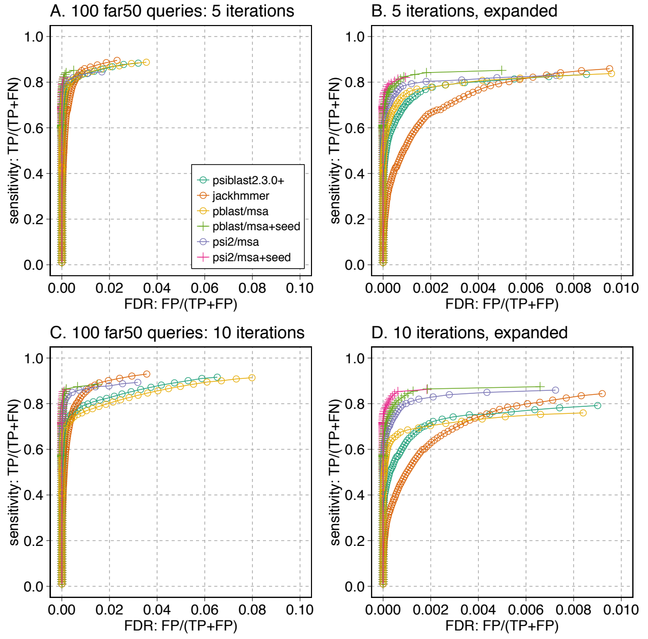

Supplementary Fig. 1. *Iterative search sensitivity before and after query seeding (far50 queries)*— Six iterative search strategies are shown on the 100 far50 embedded queries: unmodified NCBI `psiblast` (psiblast2.3.0+, ○), `jackhmmer` (jackhmmer, ○), `psiblast` using the `msa` strategy outlined in Fig. 1, (pblast/msa, ○), and together with query seeding (pblast/msa+seed,+), and `psisearch2` without query seeding (psi2/msa, ○), and with query seeding (cpsi2,+).

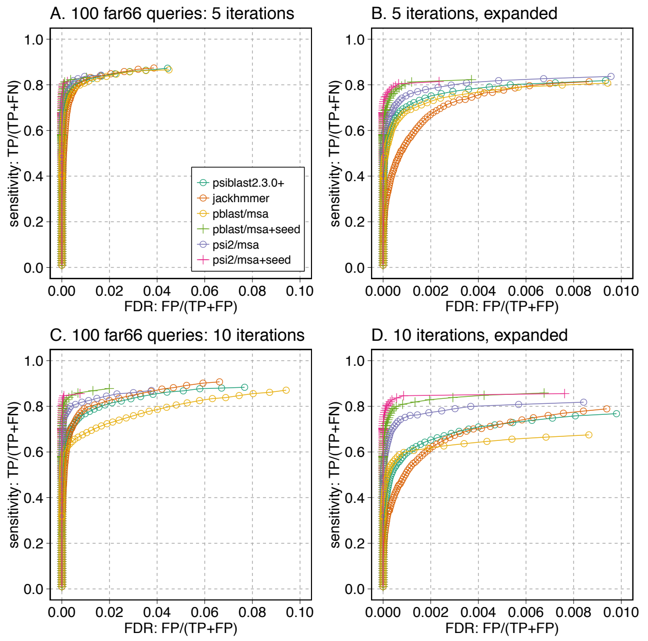

Supplementary Fig. 2. *Iterative search sensitivity before and after query seeding (far66 queries)*— Six iterative search strategies are shown on the 100 far66 embedded queries: unmodified NCBI `psiblast` (psiblast2.3.0+, ○), `jackhmmer` (jackhmmer, ○), `psiblast` using the `msa` strategy outlined in Fig. 1, (pblast/msa, ○), and together with query seeding (pblast/msa+seed, +), and `psisearch2` without query seeding (psi2/msa, ○), and with query seeding (cpsi2,+).

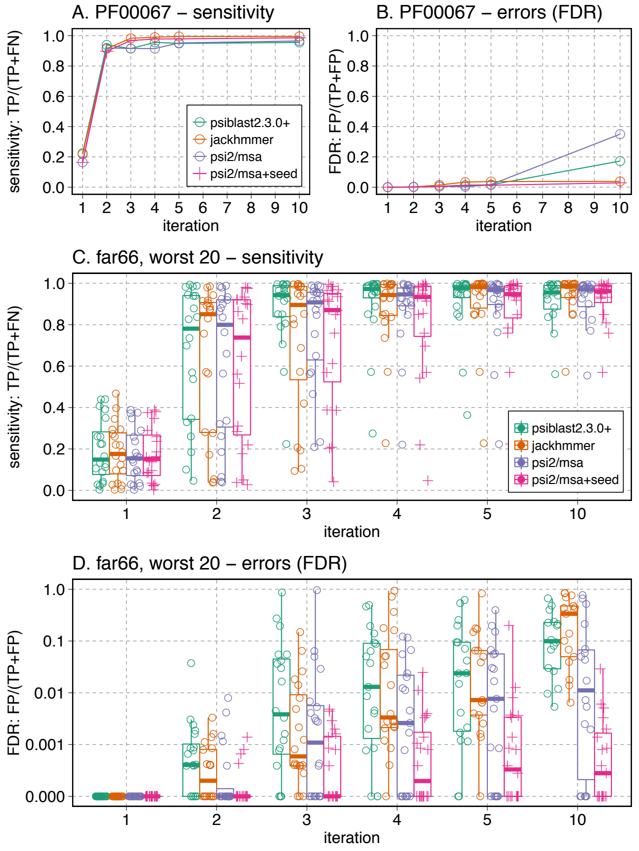

Supplementary Fig. 3. Sensitivity and selectivity (FDR) for PF00067 (A,B) and 20 challenging far66 queries (C,D). The distribution of the sensitivity (A,C; fraction of true positives found) and FDR (B, D) are shown for four different search strategies, NCBI `psiblast` (○), `jackhmmer` (○), `psisearch2` without query seeding (○), and `psisearch2` with query seeding (+). The boxplots show the median, first and third quartiles, and 1.5 times the inter-quartile range. FDR (panel D) is plotted on a log scale. The median bar is never visible in the `psisearch2` seeded box because fewer than half the challenging families have false-positives.

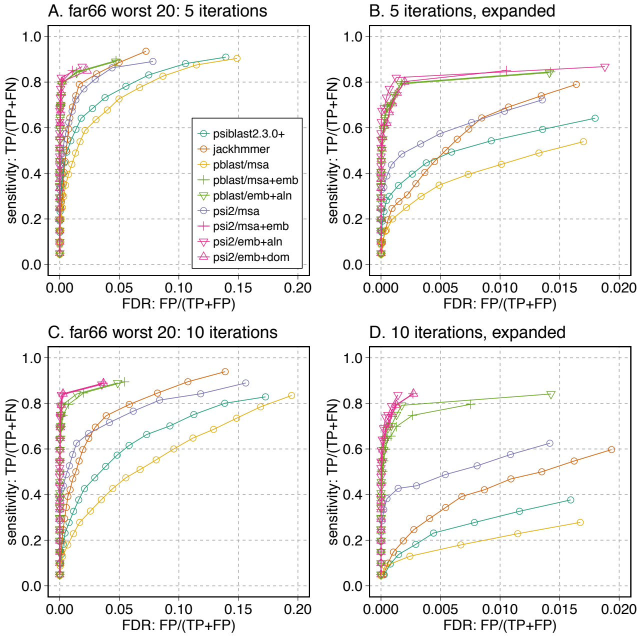

Supplementary Fig. 4. Comparison of seeding and over-extension control strategies (far66 queries). Nine iterative search strategies are shown on the 20 most challenging queries from the far66 set. In addition to the six strategies shown in Fig. 3, three combinations of two over-extension limiting strategies are shown: (1) `psiblast/msa` with alignment extension limited by alignment history (pblast/emb+aln, ▽; (2) `psisearch2` with seeding limited by alignment history (psi2/emb+aln, ▽), and (3) `psisearch2` seeded with domain boundaries (psi2/emb+dom, △). Results after five (A,B) or ten (C, D) iterations.

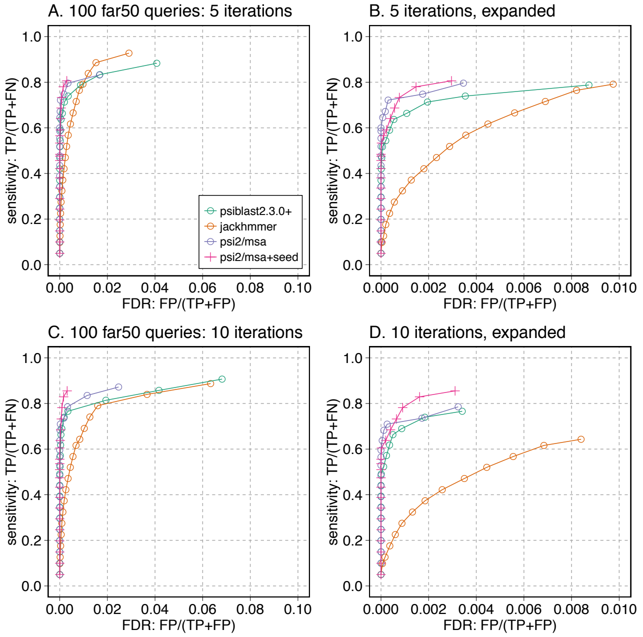

Supplementary Fig. 5. Six iterative search strategies are shown on unembedded versions of the 20 challenging far50 queries: unmodified NCBI `psiblast` (psiblast2.3.0+, ○), `jackhmmer` (jackhmmer, ○), `psiblast` using the `msa` strategy outlined in Fig. 1, (pblast/msa, ○), and together with query seeding (pblast/msa+seed, +), and `psisearch2` without query seeding (psi2/msa, ○), and with query seeding (cpsi2, +). Results after five (A,B) or ten (C, D) iterations.

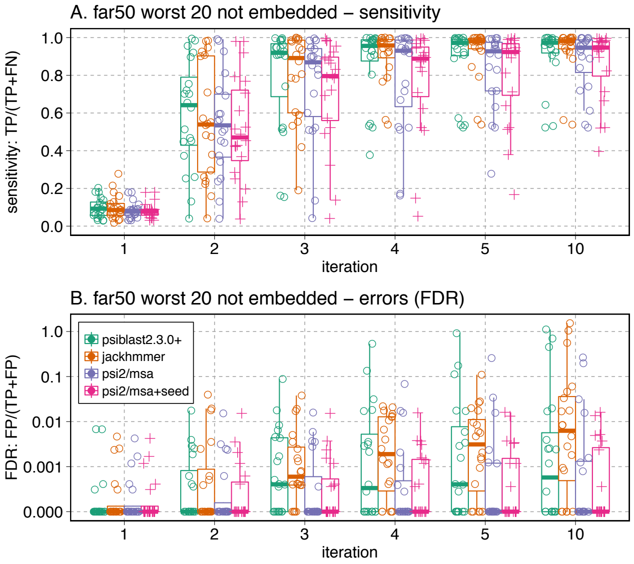

Supplementary Fig. 6. *Sensitivity (A) and FDR (B) for non-embedded queries from the far50 set*— The distribution of the sensitivity (A; fraction of true positives found) and FDR (B) are shown for four different search strategies, NCBI `psiblast` (○), `jackhmmer` (○), `psisearch2` without query seeding (○), and `psisearch2` with query seeding (+). The boxplots show the median, first and third quartiles, and 1.5 times the inter-quartile range. FDR (panel D) is plotted on a log scale.

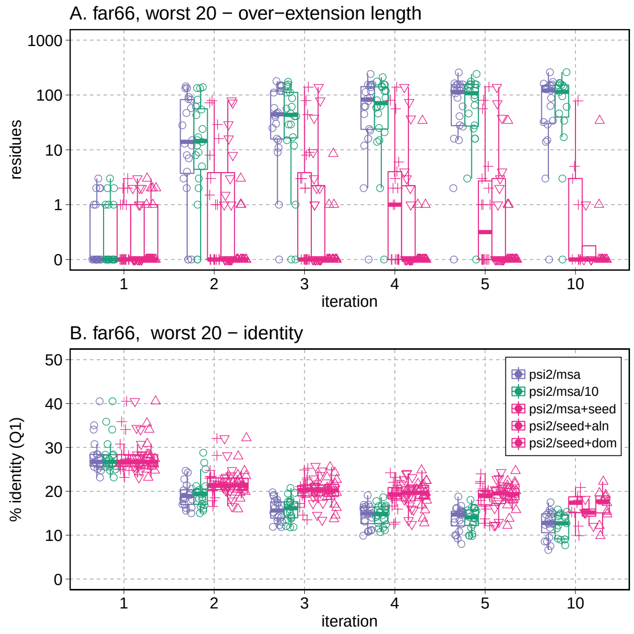

Supplementary Fig. 7. Query seeding reduces over-extension and increases alignment identity (far66 queries). The distributions of the median over-extension (A) and the bottom quartile of percent identity (B) for all alignments with $E() < 0.001$ for the 20 challenging far66 queries is shown. Symbols and colors are as in Fig. 6.

Supplementary Table 1. False discovery rates (FDR) and sensitivity (maximum family coverage) for 100 embedded far66 queries

Program	Iter.	F50%^{a}	FDR 80%^{b}	FDR Max.^{c}	Sens.^{d}
psiblast	it1	–	–	**0.0000**	0.1614
	it5	0.0001	0.0053	0.0444	0.8728
	it10	0.0003	0.0166	0.0767	0.8834
jackhmmer	it1	–	–	0.0076	**0.1688**
	it5	0.0007	0.0065	0.0387	**0.8738**
	it10	0.0009	0.0118	0.0662	**0.9073**
psisearch2/ msa	it1	–	–	**0.0000**	0.1447
	it5	**0.0000**	0.0029	0.0164	0.8425
	it10	**0.0000**	0.0038	0.0376	0.8695
psisearch2/ msa+seed	it1	–	–	**0.0000**	0.1444
	it5	**0.0000**	**0.0006** 5.0X	**0.0024** 7.0X	0.8143
	it10	**0.0000**	**0.0001** 26X	**0.0076** 4.9X	0.8558
psiblast/ msa	it1	–	–	0.0000	0.1614
	it5	0.0001	0.0078	0.0450	0.8656
	it10	0.0002	0.0457	0.1125	0.8804
psiblast/ msa+seed	it1	–	–	**0.0000**	0.1614
	it5	**0.0000**	0.0009 8.9X	0.0103 4.4X	0.8324
	it10	**0.0000**	0.0006 79X	0.0199 5.7X	0.8780

^{a}False discovery rate (FDR) at 50% weighted family coverage after 5 or 10 iterations (Iter.). ^{b}FDR at 80% family coverage. ^{c}Maximum FDR. ^{d}Maximum weighted family coverage. The best performing method (lowest FDR, highest sensitivity) is shown in **bold**. **X** values show the reduction in FDR compared with unseeded MSAs for `psiblast` and `psisearch2`.

Supplementary Table 2. False discovery rates (FDR) and sensitivity (maximum family coverage) for 100 full-length far50 queries

Program	Iter.	F50%^{a}	FDR 80%^{b}	FDR Max.^{c}	Sens.^{d}
psiblast	1	–	–	**0.0027**	0.1852
	5	**0.0000**	**0.0009**	0.0184	0.8860
	10	**0.0000**	0.0029	0.0522	0.9183
jackhmmer	1	–	–	0.0065	**0.2084**
	5	0.0004	0.0024	0.0189	**0.9186**
	10	0.0004	0.0025	0.0385	**0.9378**
psisearch2/ msa	1	–	–	0.0032	0.1691
	5	**0.0000**	0.0013	0.0152	0.8524
	10	**0.0000**	**0.0003**	0.0205	0.8918
psisearch2/ msa+seed	1	–	–	0.0032	0.1689
	5	**0.0000**	0.0054 0.2X	0.0077 2.0X	0.8062
	10	**0.0000**	0.0006 0.5X	0.0081 2.5X	0.8460
psiblast/ msa	1	–	–	**0.0027**	0.1852
	5	**0.0000**	0.0011	0.0230	0.8920
	10	**0.0000**	0.0027	0.0507	0.9179
psiblast/ msa+seed	1	–	–	**0.0027**	0.1852
	5	**0.0000**	0.0012 1.0X	**0.0071** 3.2X	0.8315
	10	**0.0000**	0.0009 2.8X	**0.0078** 6.5X	0.8544

^{a}False discovery rate (FDR) at 50% weighted family coverage after 5 or 10 iterations (Iter.). ^{b}FDR at 80% family coverage. ^{c}Maximum FDR. ^{d}Maximum weighted family coverage. The best performing method (lowest FDR, highest sensitivity) is shown in **bold**. **X** values show the reduction in FDR compared with unseeded MSAs for `psiblast` and `psisearch2`. **X** values show the reduction in FDR compared with unseeded MSAs for `psiblast` and `psisearch2`.

Supplementary Table 3. False discovery rates (FDR) and sensitivity (maximum family coverage) for 100 full-length far66 queries

Program	Iter.	F50%^{a}	FDR 80%^{b}	FDR Max.^{c}	Sens.^{d}
psiblast	1	–	–	**0.0020**	0.2170
	5	**0.0000**	0.0059	0.0412	0.8812
	10	**0.0000**	0.0088	0.0648	0.9021
jackhmmer	1	–	–	0.0055	**0.2364**
	5	0.0006	0.0042	0.0367	**0.9125**
	10	0.0005	0.0043	0.0690	**0.9362**
psisearch2/ msa	1	–	–	0.0024	0.2009
	5	**0.0000**	0.0033	0.0278	0.8453
	10	**0.0000**	0.0013	0.0375	0.8797
psisearch2/ msa+seed	1	–	–	0.0024	0.2013
	5	**0.0000**	0.0024 1.4X	**0.0070** 4.0X	0.8119
	10	**0.0000**	**0.0004** 3.6X	**0.0095** 4.0X	0.8496
psiblast/ msa	1	–	–	**0.0020**	0.2170
	5	**0.0000**	0.0057	0.0440	0.8799
	10	**0.0000**	0.0092	0.0634	0.8964
psiblast/ msa+seed	1	–	–	**0.0020**	0.2170
	5	**0.0000**	**0.0023** 2.5X	0.0131 3.4X	0.8383
	10	**0.0000**	0.0025 3.7X	0.0212 3.0X	0.8651

^{a}False discovery rate (FDR) at 50% weighted family coverage after 5 or 10 iterations (Iter.). ^{b}FDR at 80% family coverage. ^{c}Maximum FDR. ^{d}Maximum weighted family coverage. The best performing method (lowest FDR, highest sensitivity) is shown in **bold**.
